# Cold Atmospheric Plasma Reduces Migration and Viability of Oral Squamous Cell Carcinoma Cells and Increases E-Cadherin Expression

**DOI:** 10.3390/cimb48090943

**Published:** 2026-09-15

**Authors:** Paola Della Monica, Federica Colapietra, Carmine Lauretta, Marzia Di Donato, Tatjana Maravic, Lorenzo Breschi, Eleonora Lo Muzio, Marina Di Domenico, Lorenzo Lo Muzio, Lucio Lo Russo, Andrea Ballini

**Affiliations:** 1 Department of Clinical and Experimental Medicine, University of Foggia, Viale Pinto, 1, 71122 Foggia, Italy; paola.dellamonica@unifg.it (P.D.M.); lorenzo.lomuzio@unifg.it (L.L.M.); lucio.lorusso@unifg.it (L.L.R.); 2 Department of Precision Medicine, University of Campania “Luigi Vanvitelli”, Via L. De Crecchio, 80138 Naples, Italy; federica.colapietra@unicampania.it (F.C.); carmine.lauretta@unicampania.it (C.L.); marzia.didonato@unicampania.it (M.D.D.); 3 Department for Biomedical and Neuromotor Sciences, University of Bologna, Via San Vitale 59, 40125 Bologna, Italy; tatjana.maravic@unibo.it (T.M.); lorenzo.breschi@unibo.it (L.B.); 4 Department of Life Science, Health and Health Professions, Link Campus University, 00165 Rome, Italy

**Keywords:** oral squamous cell carcinoma, cold atmospheric plasma, head and neck cancer, cell migration, cell proliferation

## Abstract

**Background:** Cold atmospheric plasma (CAP) has emerged as a promising experimental anticancer strategy, yet its influence on oral squamous cell carcinoma (OSCC) migration and viability remains moderately understood. The aim of this workpaper was to examine how CAP exposure influences wound closure, WST-1 metabolic signal, and key proteins involved in epithelial adhesion and cell-cycle regulation in OSCC models. **Methods:** HSC-3 and CAL27 head and neck squamous cell carcinoma (HNSCC) cells were exposed to CAP generated via Piezoelectric Direct Discharge (PDD) technology for 20 s under standardized conditions. Wound closure was assessed by wound-healing assay under proliferation-restricted conditions (50 μM Ara-C), while relative cellular metabolic activity was measured using WST-1. E-cadherin, N-cadherin, and Cyclin A were evaluated by Western blot. Publicly available baseline transcriptomic data were additionally analyzed to characterize migration- and cytoskeleton-related expression patterns in the two HNSCC models. These transcriptomic data were independent of CAP treatment. **Results:** A 20 s CAP exposure significantly delayed wound closure in both HNSCC cell lines under proliferation-restricted conditions and reduced the WST-1 signal. Cyclin A levels also decreased, supporting an effect on cell growth and cell-cycle regulation, although the available data do not establish cell-cycle arrest. E-cadherin increased after CAP treatment, whereas N-cadherin remained low without a statistically significant change. Baseline transcriptomic mapping further showed differences between HSC-3 and CAL27 in genes related to actin-cytoskeleton organization, focal adhesion, and migration. These data were used to contextualize the experimental phenotype and were not interpreted as CAP-induced transcriptional changes. **Conclusions:** In these in vitro OSCC models, CAP exposure was associated with slower wound closure, lower WST-1 signal, increased E-cadherin, and reduced Cyclin A expression. These findings suggest further investigation of CAP as a potential modulator of OSCC cellular metabolic activity, adhesion-related markers, and cell-cycle-associated proteins.

## 1. Introduction

Cold atmospheric plasma (CAP) is a non-thermal technology that has attracted considerable interest in oncology [1]. Experimental studies indicate that CAP can affect tumor-cell survival and proliferation and, under some conditions, may exert more limited effects on non-neoplastic cells [1,2,3,4]. CAP is a partially ionized gas generated at atmospheric pressure and near room temperature. Its biologically active components include reactive oxygen and nitrogen species (ROS/RNS), charged particles, transient electric fields, and ultraviolet radiation [2]. Together, these factors can disrupt cellular redox homeostasis and engage stress-response pathways that influence cell fate [3]. The same properties have encouraged the use of CAP in antimicrobial applications, wound healing, and tissue regeneration [4,5]. In cancer models, CAP exposure has been associated with oxidative stress, mitochondrial dysfunction, apoptosis, and reduced cell growth [6]. Tumor cells may be particularly susceptible to this oxidative challenge because they often exhibit elevated basal oxidative stress and altered antioxidant defenses [7]. CAP has also been reported to affect cell-cycle regulators, including cyclins and cyclin-dependent kinases [6]. Cyclin A is especially relevant in this context because of its role in DNA replication and cell-cycle progression [8,9]. However, although several studies have investigated the effects of CAP on tumor-cell survival and proliferation, its influence on wound closure and related cellular responses remains incompletely characterized, particularly in oral squamous cell carcinoma (OSCC) models [3].

Cell migration and wound closure involve coordinated processes including the actin cytoskeleton remodeling, the turnover of focal adhesions, and the dynamic cell–matrix interactions. Rho-family GTPases, focal adhesion kinase (FAK), integrins, and other cytoskeleton-associated proteins are key components of this regulatory network [10]. Because redox signaling can influence several of these pathways, CAP-derived reactive species may plausibly affect cellular behaviors associated with wound closure and migration [11]. However, the molecular and functional mechanisms underlying these effects remain only partly defined, particularly in OSCC [3]. OSCC is locally aggressive malignancy that frequently spreads to regional lymph nodes, making the identification of strategies capable of modulating tumor-cell growth and invasive behavior of considerable interest [12]. However, conventional wound-healing assays do not strictly distinguish physical cell movement from other processes contributing to gap closure, including cell proliferation and changes in cellular fitness. Consequently, impaired wound closure cannot necessarily be interpreted as evidence of a migration-specific effect [10].

In the present study, we investigated the effects of a standardized 20 s CAP exposure in two human OSCC cell models with distinct biological characteristics and origins: HSC-3, derived from a lymph node metastasis, and CAL27, derived from a primary tongue lesion. Wound-healing assays were performed under Ara-C-mediated proliferation-restricted conditions to limit the contribution of cell division to gap closure. In parallel, WST-1 measurements were used to assess relative cellular metabolic activity, while Cyclin A expression was evaluated as a cell-cycle-associated marker. E-cadherin and N-cadherin were examined as adhesion-related markers associated with epithelial and mesenchymal cellular phenotypes. To further characterize the molecular background of the two cell models, we additionally analyzed publicly available baseline transcriptomic datasets, focusing on genes associated with actin-cytoskeleton organization, focal adhesion, and migration-related pathways. Importantly, these datasets represent untreated baseline cellular states and were not used to infer CAP-induced transcriptional responses or dynamic pathway regulation. Instead, the transcriptomic analysis was included to provide descriptive molecular context for the experimental findings and to highlight pre-existing differences between HSC-3 and CAL27.

By combining wound-healing assays performed under proliferation-restricted conditions with the assessment of WST-1 signal, adhesion-related proteins, Cyclin A expression, and baseline transcriptomic profiling of cytoskeleton- and migration-related pathways, this study provides a multi-level characterization of the cellular responses associated with a standardized Piezoelectric Direct Discharge (PDD)-based CAP exposure in two biologically distinct OSCC models.

## 2. Materials and Methods

### 2.1. Cell Culture

Human OSCC cell lines HSC-3 (JCRB0623; JCRB Cell Bank, National Institute of Biomedical Innovation, Health and Nutrition, Ibaraki, Osaka, Japan) and CAL27 (CRL-2095; American Type Culture Collection [ATCC], Manassas, VA, USA) were selected as representative in vitro models of head and neck squamous cell carcinoma (HNSCC). Both cell lines originate from human tongue squamous cell carcinoma, the most common anatomical site of OSCC [13]. The two models were selected to investigate the cellular responses to CAP across distinct biological backgrounds. Specifically, HSC-3 was derived from a lymph node metastasis and is generally characterized by a more aggressive and invasive phenotype, whereas CAL27 was derived from a primary lesion and represents a moderately differentiated OSCC model [14]. In addition, publicly available baseline transcriptomic data indicate differences between the two cell lines in the expression of genes associated with cytoskeletal organization, focal adhesion, and migration-related pathways. These baseline molecular differences were considered as descriptive context for the interpretation of the experimental findings. The study was specifically designed to characterize CAP-associated responses across biologically distinct malignant OSCC backgrounds rather than to evaluate tumor-versus-normal cell selectivity; therefore, non-neoplastic oral epithelial cells were not included in the original experimental design.

Reference short tandem repeat (STR) profiles for both cell lines are available from the respective repositories [13]. To limit phenotypic drift during prolonged in vitro culture, all experiments were performed with low-passage cells maintained below passage 10, in line with previously published protocols using HSC-3 and CAL27 cells. Cells were cultured in high-glucose Dulbecco’s Modified Eagle Medium (DMEM, Microgem S.r.l., Naples, Italy; cat. no. PMSTVMSV-500 mL) supplemented with 10% fetal bovine serum (FBS, GIBCO, Thermo Fisher Scientific, Waltham, MA, USA) and 1% penicillin-streptomycin (GIBCO, Thermo Fisher Scientific, Waltham, MA, USA), at 37 °C in a humidified atmosphere containing 5% CO_2_. Cultures were routinely subcultured at 70–80% confluence and tested for mycoplasma contamination [15].

### 2.2. Cold Atmospheric Plasma Protocol

CAP treatment was performed using the Relyon Plasma PZ3 handheld device (Model BA-PZ3_ML/Part No. F0354402; Relyon Plasma GmbH, Regensburg, Germany), which operates using Piezoelectric Direct Discharge (PDD^®^) technology. Prior to the study, the device was calibrated and validated according to manufacturer protocols to ensure operational reproducibility across experimental runs. The device has a maximum nominal power input of 18 W, an operating frequency of approximately 50 kHz, and an internal peak-to-peak output voltage of approximately 15 kV. Ambient air was used as the working gas at the default factory output level (100%), with the integrated micro-fan generating a continuous gas flow of approximately 10–18 L/min. Unless otherwise specified, the Near Needle applicator nozzle was used, producing a plasma plume 5–15 mm in effective length and 15–29 mm in effective exposure diameter.

Treatments were conducted under controlled atmospheric conditions at a room temperature of 22 ± 2 °C and a relative humidity of 45–55%. To standardize experimental geometry, the nozzle was positioned at a fixed distance of 10 mm above the liquid surface of the culture medium, perpendicular to the liquid layer. The treatment distance was consistently checked and maintained throughout the experiments. Media volume was standardized across all multiwell formats (100 µL per well in 96-well plates; 500 µL per well in 24-well plates) to maintain consistent liquid column depth and treatment conditions. The plasma plume was moved in a standardized raster pattern across each well to ensure uniform exposure and minimize localized heating or evaporation. Direct contact with the liquid interface was avoided. Cells received direct CAP exposure for exactly 20 s per well.

Although direct liquid-phase ROS/RNS measurements and a dedicated sham airflow control were not incorporated into the primary workflow, all experiments were performed using the same standardized device settings, treatment geometry, exposure distance, medium volume, and exposure duration to maximize experimental consistency across biological replicates. The absence of direct ROS/RNS quantification and of a sham airflow control is acknowledged as a limitation of the study.

To assess potential thermal effects, medium temperature was monitored using a non-contact infrared thermometer. Under the experimental conditions used, the temperature variation during the 20 s CAP exposure did not exceed 1.5 °C, and the temperature of the medium remained below 24 °C.

Untreated cells, kept under the same environmental conditions for the same duration without device activation, served as controls. At least three independent biological replicates were performed under the reported exposure conditions.

### 2.3. Cell Viability Determination

Relative metabolic activity and viable cell mass were assessed using the WST-1 colorimetric assay (cat. no. 5015944001; Sigma-Aldrich, St. Louis, MO, USA), as previously described [16]. Cells were seeded in 96-well plates at 5 × 10^3^ cells in 100 μL of complete medium per well, exposed to CAP for 20 s or left untreated, and incubated for 24 or 48 h. WST-1 reagent was then added at a 1:10 dilution and incubated for 2 h at 37 °C according to the manufacturer’s instructions. Absorbance was measured at 450 nm using a Spark microplate reader (Tecan, Männedorf, Switzerland). Because WST-1 measures NAD(P)H-dependent mitochondrial dehydrogenase activity, it reflects metabolic activity and viable cell mass rather than proliferation directly; the results were expressed as relative WST-1 signal compared with untreated controls.

### 2.4. Wound Healing Assay

Wound closure was assessed using a wound-healing (scratch) assay. HNSCC cells were seeded in 24-well plates at a density of 2 × 10^5^ cells/well in 500 μL of complete medium and allowed to reach full confluence (approximately 24 h). A linear scratch was generated across the confluent cell monolayer using a sterile 200 μL pipette tip to create a uniform cell-free gap, and detached cells were removed by two gentle washes with phosphate-buffered saline (PBS) [17].

**Figure 1:**
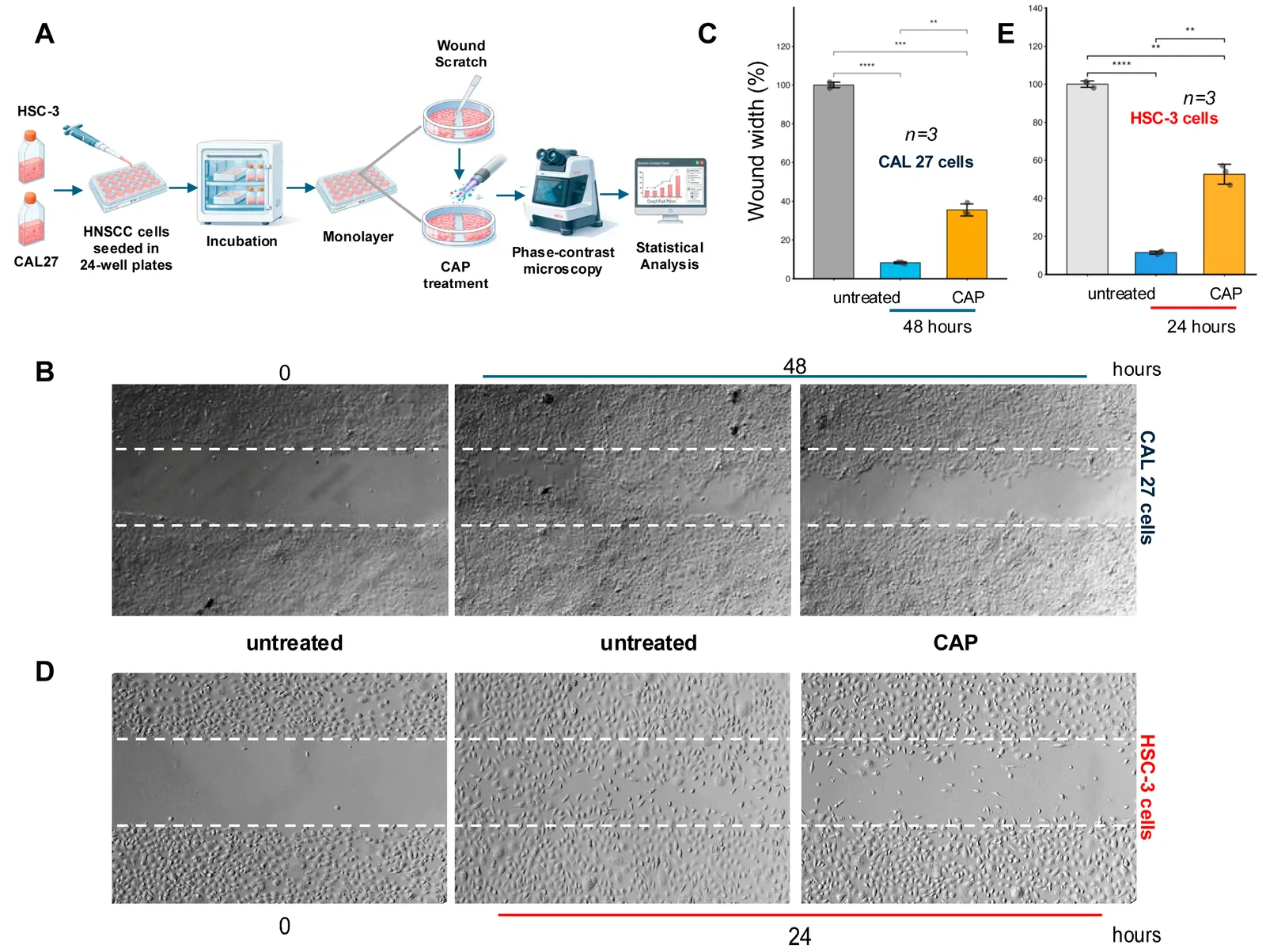
**Cold atmospheric plasma (CAP) exposure reduces wound closure in HNSCC cell lines.** (**A**) Schematic representation of the experimental workflow for the wound healing assay, including cell seeding, scratch induction, CAP treatment, image acquisition, and quantitative analysis. (**B**,**D**) Representative phase-contrast images of wound-healing assays performed in (**B**) CAL27 and (**D**) HSC-3 cells. White dashed lines indicate the wound margins. To account for differences in basal closure kinetics, the endpoint was 48 h for CAL27 cells and 24 h for HSC-3 cells. Images show untreated controls and CAP-exposed cells at the indicated time points. (**C**,**E**) Quantification of wound closure in (**C**) CAL27 and (**E**) HSC-3 cells, calculated as [(A_0_ − A_t_)/A_0_] × 100, where A_0_ is the wound area at time 0 and A_t_ is the wound area at the indicated time point. Bar graphs represent mean ± SD from three independent biological replicates (*n* = 3). Bar colors represent the different experimental time points and treatments applied to the cells: the gray bar indicates the quantification of wound closure for the untreated controls at time 0 h; the blue bar represents the quantification of wound closure for the untreated controls at 48 h for CAL27 cells and 24 h for HSC-3 cells; the orange bar represents the quantification of wound closure following CAP treatment at 48 h for CAL27 cells and 24 h for HSC-3 cells. Statistical significance was determined by one-way ANOVA followed by Holm–Šídák’s multiple-comparisons test, comparing each treatment group with the untreated control (*p* < 0.05, ** *p* < 0.01, *** *p* < 0.001, **** *p* < 0.0001).

To limit the contribution of cell proliferation to wound closure, cells were maintained throughout the assay in 50 μM cytosine β-D-arabinofuranoside (Ara-C; Sigma-Aldrich, St. Louis, MO, USA), a specific inhibitor of DNA synthesis. This concentration was selected based on established HNSCC protocols to suppress cell division while allowing wound closure to be monitored over the experimental timeframe. Immediately after scratching, cells were exposed to CAP for 20 s. Scratch closure was monitored at defined timepoints using phase-contrast microscopy, acquiring images at 0 h and at either 24 or 48 h, as specified in Figure 1, using a Leica MICA Microhub live-cell imaging system (Leica Microsystems, Wetzlar, Germany) and processed with Leica Application Suite X software (LAS X, version 6.2.2.28360.1, Leica Microsystems, Wetzlar, Germany).

Different endpoint durations were selected based on the distinct wound-closure kinetics observed for the two cell lines under the experimental conditions used. Untreated HSC-3 control cultures reached complete gap closure within 24 h, whereas CAL27 control cultures required 48 h to reach the corresponding endpoint. The cell-line-specific observation windows were therefore selected to evaluate the effects of CAP within the respective wound-closure timeframe of each model.

Untreated control monolayers of both cell lines achieved complete (100%) gap closure at their respective endpoints, indicating that, under the experimental conditions used, the selected Ara-C concentration did not prevent wound closure over the duration of the assay. However, because the scratch assay reflects a composite process involving cellular movement and other aspects of cellular fitness, these observations were not interpreted as evidence of an isolated migration-specific effect.

Wound area was measured in multiple fields per well using ImageJ (National Institutes of Health, Bethesda, MD, USA) with the MRI Wound Healing Tool plugin. Measurements from independent experiments were averaged, and wound closure was calculated relative to the initial wound area at T0.

The percentage of wound closure was calculated according to the following formula:

Wound closure (%) = [(A_0_ − A_t_)/A_0_] × 100

where A_0_ represents the wound area at time 0 and A_t_ represents the wound area at the indicated time point. Values were calculated for each field analyzed and averaged to obtain the final percentage of wound closure for each experimental condition.

### 2.5. Western Blot Analysis

Cells were lysed in ice-cold lysis buffer containing 50 mM Tris-HCl (pH 7.4), 150 mM NaCl, 1% Triton X-100, 1 mM EDTA, 5 mM MgCl_2_, and 1 mM EGTA, supplemented with protease and phosphatase inhibitors (1 mM phenylmethylsulfonyl fluoride (PMSF), protease inhibitor cocktail, and sodium orthovanadate). Cell lysates were clarified by centrifugation, and protein concentrations were determined using the Bio-Rad protein assay (Bio-Rad Laboratories, Hercules, CA, USA).

Equal amounts of protein (30 μg) were separated by 11% SDS–polyacrylamide gel electrophoresis (SDS–PAGE) and electrotransferred onto nitrocellulose membranes. Membranes were blocked with 5% bovine serum albumin (BSA) in Tris-buffered saline containing 0.1% Tween-20 (TBS-T) and incubated overnight at 4 °C with primary antibodies diluted 1:1000, including anti-E-cadherin (E-AB-31261, Elabscience, Wuhan, China), anti-N-cadherin (E-AB-70061, Elabscience, Wuhan, China), and anti-Cyclin A Polyclonal Antibody (E-AB-90874, Elabscience, Wuhan, China).

After washing, membranes were incubated with appropriate horseradish peroxidase (HRP)-conjugated secondary antibodies (Thermo Fisher Scientific, Waltham, MA, USA) diluted 1:2000, and immunoreactive bands were visualized using an enhanced chemiluminescence (ECL) detection system (Pierce ECL Western Blotting Substrate, Thermo Fisher Scientific, cat. no. 10590624).

Protein expression was normalized to GAPDH (Rabbit anti-GAPDH polyclonal antibody, cat. no. E-AB-40337; Elabscience Biotechnology Co., Ltd., Wuhan, China) diluted 1:1000, used as the internal loading control. When indicated, band intensities were quantified by densitometric analysis using ImageJ software (version 1.54s 26 March 2026, National Institutes of Health, Bethesda, MD, USA; https://imagej.net/ij/, accessed on 13 July 2026), and protein expression levels were expressed relative to GAPDH.

### 2.6. Transcriptomic Data Acquisition and Targeted Expression Profiling

Baseline RNA-sequencing (RNA-seq) data for HSC-3 (DepMap ID: ACH-000778) and CAL27 (DepMap ID: ACH-000832) were obtained from the Cancer Dependency Map (DepMap; Public 24Q2 release). Gene-expression values, reported as log_2_(TPM + 1), were used to describe the baseline, untreated molecular profile of each model. We examined a focused panel of genes involved in cell adhesion, focal adhesion, cytoskeletal organization, extracellular-matrix remodeling, and migration-associated signaling, including ITGB1, LAMC2, VIM, RHOA, PXN, ITGA3, RAC1, LAMA3, PTK2, MMP2, and MMP9. Expression levels were displayed as horizontal bar plots generated with the ggplot2 package in R.

This targeted analysis was performed to provide descriptive molecular context for the experimental findings and to highlight pre-existing differences between the two cell models. It was not designed as an unbiased transcriptome-wide enrichment analysis and, importantly, because the datasets represent untreated baseline cellular states, it was not used to infer CAP-induced transcriptional responses or dynamic pathway regulation.

### 2.7. Descriptive Topological Mapping of the Actin Cytoskeleton Pathway

Gene identifiers were converted to Entrez IDs using the org.Hs.eg.db R/Bioconductor package (version 3.20.0; https://bioconductor.org/packages/org.Hs.eg.db, accessed on 13 July 2026). For each cell line, expression values were normalized to the mean expression of the HNSCC cell-line cohort in DepMap, generating lineage-adjusted log_2_ fold-change (log_2_FC) values. These values were then mapped onto the KEGG ‘Regulation of Actin Cytoskeleton’ pathway (hsa04810) with the pathview R package. In the resulting maps, red indicates positive relative expression (log_2_FC > 0) and green indicates negative relative expression (log_2_FC < 0) compared with the HNSCC cohort mean. The resulting maps were used exclusively as a visual representation of relative baseline expression across pathway nodes and were not interpreted as a formal enrichment analysis, evidence of pathway activation, or evidence of CAP-induced regulation.

### 2.8. Statistical Analysis

All experiments included at least three independent biological replicates, and quantitative data are reported as mean ± standard deviation (SD). Statistical analyses were performed using GraphPad Prism software (version 9.0.0, GraphPad Software, San Diego, CA, USA). For comparisons between two groups, two-tailed Student’s *t*-tests were used. For experiments involving multiple groups, statistical significance was determined by one-way analysis of variance (ANOVA), followed by Holm–Šídák’s multiple-comparisons test, with each treatment group compared with the untreated control. A *p* value < 0.05 was considered statistically significant.

## 3. Results

### 3.1. CAP Exposure Reduces Wound Closure in HNSCC Cells

We first examined whether CAP exposure altered wound closure in CAL27 and HSC-3 cells (Figure 1A).

Confluent monolayers were scratched and treated with CAP according to the protocol described above, while Ara-C was maintained throughout the experiment to limit proliferation-related gap closure. Untreated cultures progressively closed the wound area, whereas CAP-treated cultures showed slower wound closure (Figure 1B,D). Quantitative analysis confirmed a significant reduction in wound closure in CAL27 cells at 48 h and in HSC-3 cells at 24 h (Figure 1C,E).

These data indicate that a 20 s CAP exposure significantly delayed wound closure in both HNSCC cell lines, even when the contribution of cell proliferation was pharmacologically limited by Ara-C. However, because CAP treatment simultaneously reduced the WST-1 metabolic signal, impaired overall cellular fitness and reduced metabolic capacity may have contributed to the observed delay in wound closure. Therefore, the scratch-assay results indicate an altered wound-closure phenotype but cannot be considered conclusive evidence of a migration-specific inhibitory mechanism.

### 3.2. Descriptive Mapping of Baseline Cytoskeleton-Related Expression in HNSCC Cells

**Figure 2:**
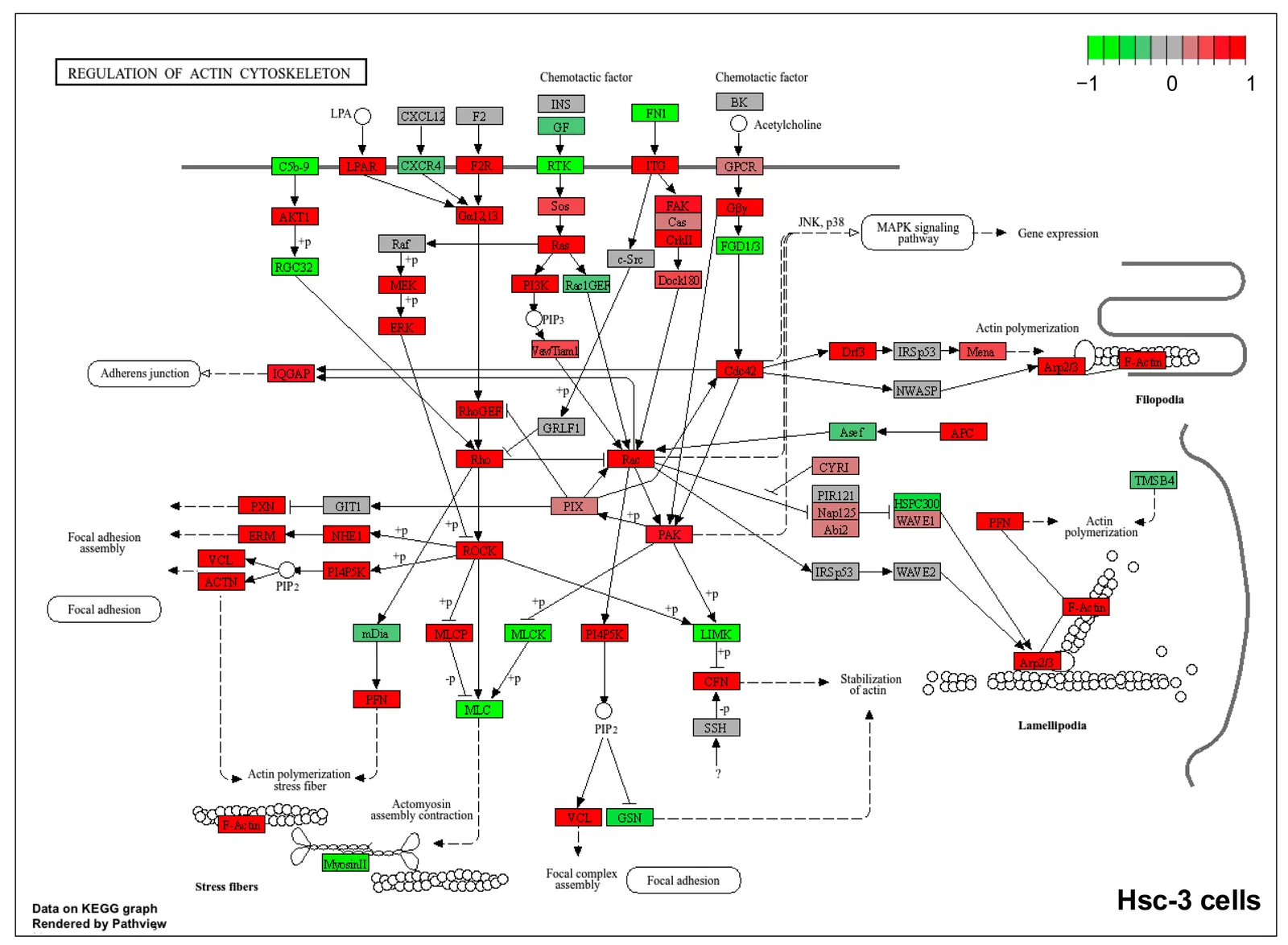
**Baseline topological expression map of the KEGG actin-cytoskeleton pathway in HSC-3 cells (hsa04810).** The KEGG ‘Regulation of Actin Cytoskeleton’ network (hsa04810) was visualized with pathview. Baseline HSC-3 transcriptomic data were normalized to the mean expression of the HNSCC cell-line cohort in DepMap Public 24Q2 and expressed as log_2_FC. Red indicates positive relative expression and green indicates negative relative expression compared with the cohort mean. The map is descriptive and is not intended to indicate pathway activation. Reproduced from the KEGG database (https://www.kegg.jp, accessed on 13 July 2026) with permission [18].

**Figure 3:**
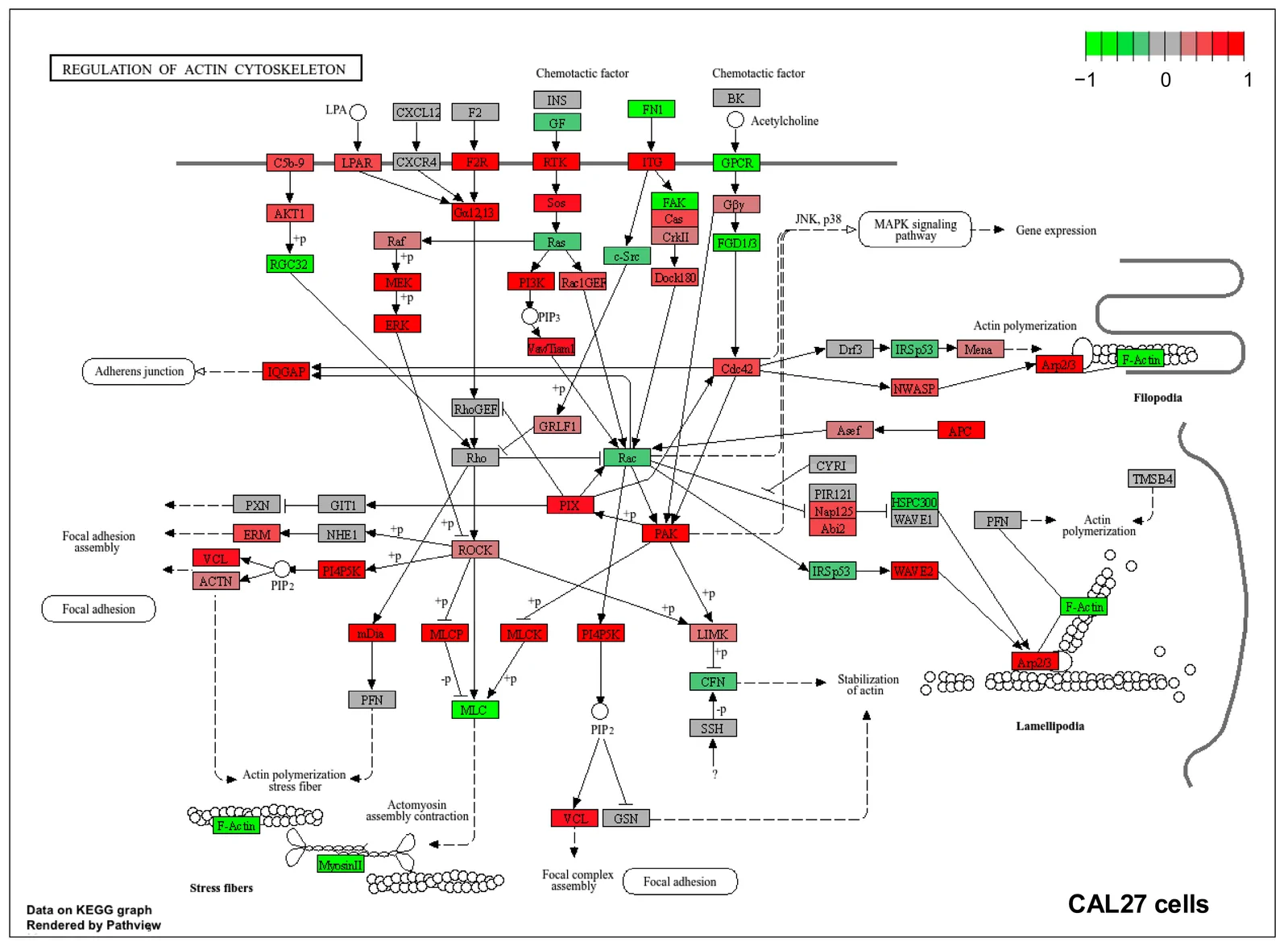
**Baseline topological expression map of the KEGG actin-cytoskeleton pathway in CAL27 cells (hsa04810).** The KEGG ‘Regulation of Actin Cytoskeleton’ pathway (hsa04810) was visualized with pathview using baseline CAL27 transcriptomic data. Expression values were normalized to the mean of the HNSCC cell-line cohort in DepMap Public 24Q2 and expressed as log_2_FC. Red indicates positive relative expression and green indicates negative relative expression compared with the cohort mean. As with the HSC-3 map, this visualization describes baseline expression and does not measure pathway activation. Reproduced from the KEGG database (https://www.kegg.jp, accessed on 13 July 2026) with permission [18].

To place the wound-healing results in a broader molecular context, baseline DepMap expression data from HSC-3 and CAL27 cells were mapped onto the KEGG “Regulation of Actin Cytoskeleton” pathway (Figure 2 and Figure 3). The resulting maps provide a cell-line-specific visualization of relative baseline expression across pathway components. They were used exclusively for descriptive comparison and do not represent a formal enrichment analysis, evidence of pathway activation, or CAP-induced pathway regulation.

Relative to the mean expression of the HNSCC cohort, HSC-3 showed both positively and negatively shifted expression across multiple genes involved in focal adhesion, integrin signaling, Rho-family GTPase signaling, and actin remodeling. This heterogeneous pattern describes the baseline molecular profile of migration- and cytoskeleton-related components in HSC-3 but does not indicate pathway activation or functional migratory activity. However, these data do not establish functional pathway activation, invasive capacity, or responsiveness to CAP.

Both HSC-3 and CAL27 expressed multiple components of Rho/Rac signaling, focal adhesion and integrin pathways, and the actin-remodeling machinery. These molecular networks are involved in processes such as cell polarity, cytoskeletal reorganization, lamellipodia formation, and focal-adhesion turnover. The pathway maps therefore provide a molecular backdrop for comparing the pre-existing molecular profiles of the two cell models, without implying direct activation or suppression of these pathways by CAP. Based on this descriptive molecular context, we next examined whether CAP exposure was associated with changes in the adhesion-related markers E-cadherin and N-cadherin.

Compared with HSC-3, CAL27 showed a different pattern of relative baseline expression across several migration- and cytoskeleton-related pathway nodes, including components associated with focal adhesion and mechanotransduction. Several regulators of cytoskeletal dynamics, including Rac-associated signaling components, also differed between the two cell lines. These differences further characterize the distinct molecular backgrounds of the two models but do not directly measure migratory activity, pathway function, or responsiveness to CAP.

Overall, the baseline transcriptomic data highlight differences in the relative expression of components belonging to a migration- and cytoskeleton-relevant signaling network between HSC-3 and CAL27. The functional significance of these differences remains to be established through direct experimental validation.

### 3.3. Expression of Cytoskeleton and Migration-Related Genes in HNSCC Cell Lines

**Figure 4:**
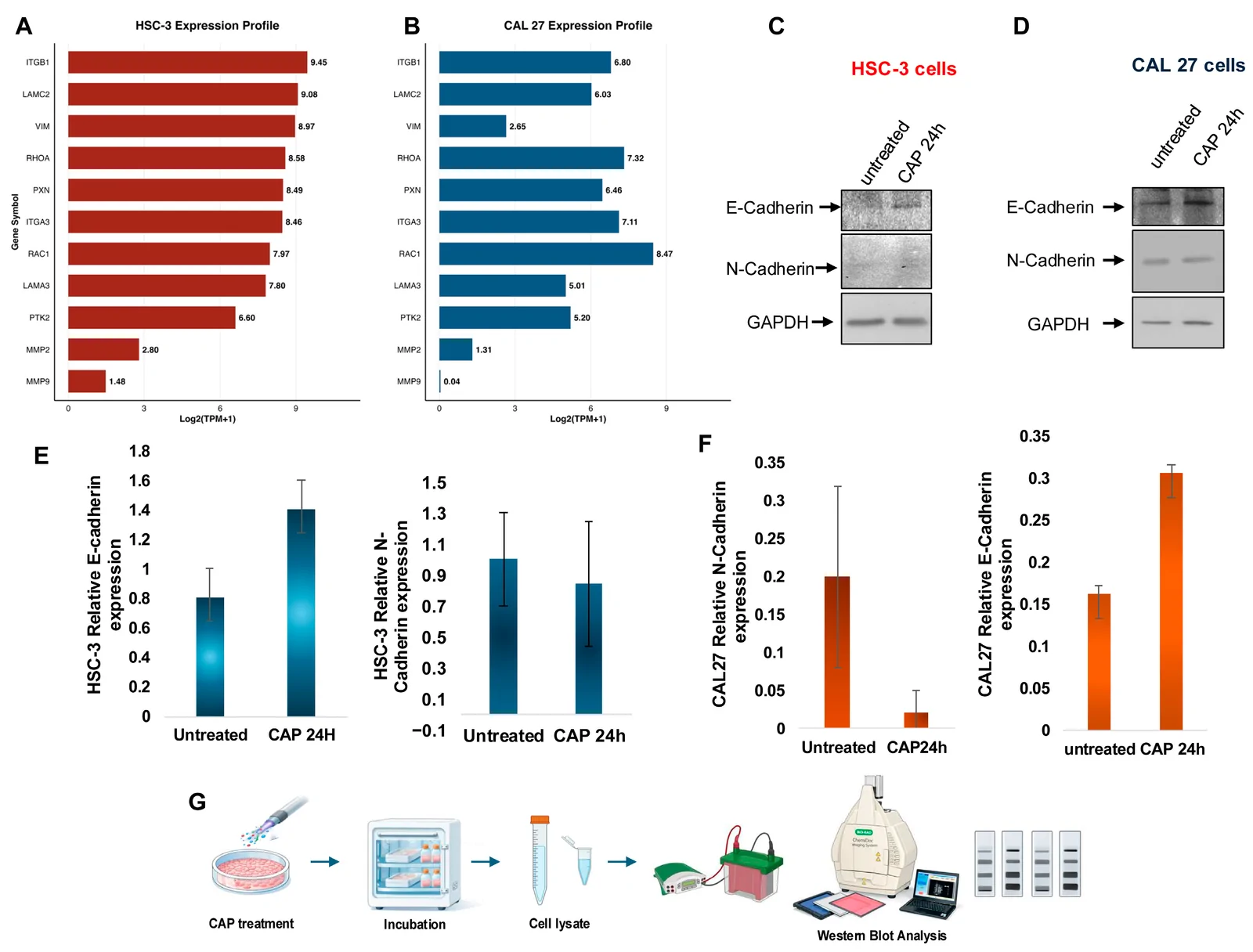
**Transcriptomic characterization and quantitative analysis of CAP-associated changes in E-cadherin and N-cadherin expression in HSC-3 and CAL27 cells.** Baseline transcriptomic profiles of (**A**) HSC-3 and (**B**) CAL27 cells. Data were obtained from the Cancer Dependency Map (DepMap; Public 24Q2 release) and represent baseline absolute expression levels [log_2_(TPM + 1)] of a targeted panel of genes associated with adhesion, focal adhesion, extracellular-matrix remodeling, and cytoskeleton dynamics. (**C**,**D**) Representative Western blots of E-cadherin and N-cadherin in (**C**) HSC-3 and (**D**) CAL27 cells under untreated conditions and 24 h after a 20 s CAP exposure, from three independent biological replicates (*n* = 3). GAPDH was used as the loading control. (**E**,**F**) GAPDH-normalized densitometric quantification of E-cadherin and N-cadherin expression represented by blue bars (HSC-3 cells, (**E**)) and orange bars (CAL27 cells, (**F**)). Data are presented as protein/GAPDH ratios. CAP exposure was associated with increased E-cadherin expression in both cell lines, whereas N-cadherin levels remained low and showed no statistically significant change following CAP exposure. (**G**) Schematic overview of the 20 s CAP exposure followed by incubation and protein analysis.

To further characterize the molecular background of the two models, we examined selected genes involved in cell adhesion, cytoskeletal organization, and migration (Figure 4A,B). Both HSC-3 and CAL27 expressed integrins (ITGB1, ITGA3), extracellular-matrix components (LAMC2, LAMA3), regulators of actin dynamics (RHOA, RAC1), and focal-adhesion-related genes including PTK2 (FAK) and PXN (paxillin). Vimentin (VIM) was also detected in both cell lines. Because VIM alone is insufficient to define an epithelial–mesenchymal transition program, it was interpreted as one element of the broader baseline profile. The transcriptomic data therefore show that adhesion- and cytoskeleton-related genes are well represented in both models, without establishing pathway activation or CAP-dependent regulation.

We then assessed E-cadherin and N-cadherin protein expression 24 h after CAP exposure. GAPDH-normalized densitometry showed higher E-cadherin levels in CAP-treated CAL27 and HSC-3 cells than in untreated controls (Figure 4C–F). N-cadherin protein levels were also quantified by GAPDH-normalized densitometric analysis. N-cadherin expression remained low in both cell lines and showed no statistically significant change following CAP exposure (Figure 4). Therefore, although CAP was associated with increased E-cadherin expression, the present data do not support the conclusion of a complete cadherin switch or a formal reversal of an epithelial–mesenchymal transition program.

The increase in E-cadherin is compatible with altered cell–cell adhesion and may contribute to the wound-closure phenotype observed after CAP treatment. However, the present data do not establish a causal relationship between E-cadherin upregulation and delayed wound closure. Likewise, the DepMap/KEGG data describe the untreated molecular background of the two models and cannot establish that specific adhesion- or cytoskeleton-related pathways mediate the CAP-associated phenotype.

### 3.4. CAP Exposure Reduces WST-1 Signal and Cyclin A Expression in HNSCC Cells

**Figure 5:**
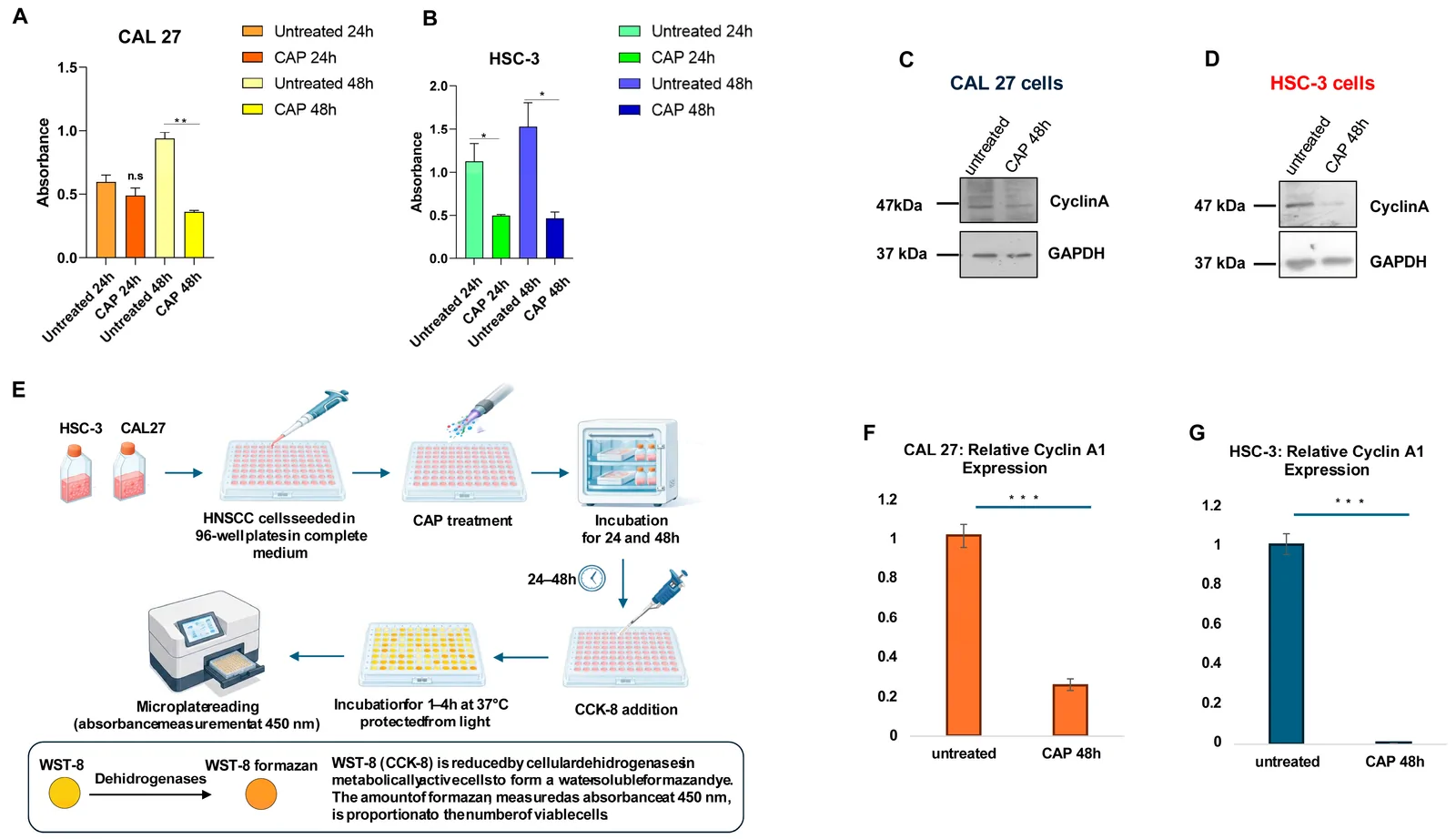
**CAP exposure reduces WST-1 signal and Cyclin A expression in HNSCC cells.** CAP exposure reduces WST-1 metabolic signal and Cyclin A expression in HNSCC cells. WST-1 absorbance in CAL27 (**A**) and HSC-3 (**B**) cells measured 24 or 48 h after a 20 s CAP exposure or in untreated controls. WST-1 absorbance at 450 nm reflects relative mitochondrial metabolic active/viable cell mass and should not be interpreted as a direct measurement of proliferation. Data are mean ± SD from three independent experiments. Statistical significance was evaluated using ANOVA (* *p* < 0.05, ** *p* < 0.01; n.s. = not significant). (**C**,**D**) Representative Western blots of Cyclin A in (**C**) CAL27 and (**D**) HSC-3 cells 48 h after a 20 s CAP exposure or in untreated controls, with GAPDH as the loading control. (**E**) Experimental workflow for 20 s CAP exposure, incubation, WST-1 reagent addition, and absorbance measurement at 450 nm. (**F**,**G**) Densitometric quantification of Cyclin A/GAPDH, shown as orange bars for CAL27 cells (**F**) and blue bars for HSC-3 cells (**G**) 48 h after CAP exposure. Data are mean ± SD (*** *p* < 0.001).

WST-1 measurements were used to assess relative metabolic activity in CAL27 and HSC-3 cells 24 and 48 h after a 20 s CAP exposure (Figure 5). CAP exposure significantly reduced the WST-1 signal in both cell lines, with a more pronounced decrease observed at 48 h. Because the WST-1 assay reflects cellular metabolic activity and is influenced by the number and metabolic state of viable cells, this reduction indicates impaired metabolic capacity and/or reduced viable cell mass but cannot distinguish among cytotoxicity, metabolic suppression, and reduced proliferation.

Cyclin A expression was likewise lower 48 h after CAP treatment (Figure 5C,D,F,G). Given the established role of Cyclin A during S-phase and G2/M progression, the observed decrease is consistent with an alteration in the expression of a cell-cycle-associated regulatory protein [19]. However, this finding alone is insufficient to demonstrate cell-cycle arrest or to identify the specific cell-cycle phase affected. Direct cell counts, DNA-synthesis assays, and flow-cytometric cell-cycle analysis would be required to resolve these possibilities.

The observed reduction in Cyclin A expression is consistent with previous reports describing CAP-associated changes in cell-cycle regulatory proteins and reduced cancer-cell growth. However, because intracellular ROS were not directly measured in the present study, a ROS-dependent mechanism cannot be established from our experimental data.

## 4. Discussion

CAP has attracted considerable attention as a non-thermal approach to cancer treatment, largely because plasma-generated reactive oxygen and nitrogen species (ROS/RNS), together with other physical components of plasma, can influence several aspects of tumor-cell biology. In a number of experimental settings, malignant cells appear to be more susceptible to these effects than their non-malignant counterparts [1]. This apparent selectivity, however, is unlikely to be universal and probably depends on both the biological model and the treatment conditions. As normal oral cells were not included in the present experiments, potential tumor selectivity was not directly assessed in this study. While the cytotoxic and growth-limiting properties of CAP have been investigated in a variety of tumor models, considerably less is known about how plasma exposure affects wound closure and migration-related cellular behavior, particularly in OSCC. Migration is a complex process that depends on continuous remodeling of cell–cell and cell–matrix contacts, actin-cytoskeleton dynamics, and focal-adhesion turnover [20,21].

CAP-treated cultures consistently showed delayed wound closure in primary (CAL27) and metastatic (HSC-3) OSCC models following a standardized 20 s exposure. Both cell lines displayed significantly less gap closure than untreated controls under proliferation-restricted conditions. To minimize the confounding contribution of cell proliferation during gap closure, 50 µM Ara-C was maintained throughout the experiment as an inhibitor of DNA synthesis. Untreated control cultures achieved complete wound closure at their respective experimental endpoints in the presence of Ara-C, indicating that, under the conditions used, Ara-C did not prevent gap closure. Therefore, CAP exposure was associated with a significant delay in wound closure under proliferation-restricted conditions. Nevertheless, wound closure cannot be considered in complete isolation from the overall condition of the cells. CAP also reduced the WST-1 signal, suggesting that lower metabolic activity and/or a reduction in viable cell mass may have contributed to the slower closure. WST-1 decrease implies that either CAP-induced metabolic suppression or sublethal fitness impairment accompanied the wound healing delay. Since active cell motility and biomechanical processes rely on intact cellular energetics and dynamic cytoskeletal turnover, reduced metabolic strength may have directly restricted the physical pace of cell movement. The most appropriate interpretation is that CAP prevented wound closure under proliferation-limited conditions, reflecting a combined cellular phenotype that involves lower metabolic capacity, alterations to adhesion-related features, and cell-cycle-associated regulation, but it does not indicate selective engagement within the migratory signaling machinery or provide conclusive evidence of a migration-exclusive pathway.

The concomitant reduction in the WST-1 signal indicates that CAP exposure may have affected overall cellular fitness in addition to processes involved in wound closure. Because active cell motility depends on adequate cellular metabolic capacity and coordinated cytoskeletal remodeling, reduced metabolic activity or viable cell mass could have contributed to the observed phenotype. The most appropriate interpretation is therefore that CAP delayed wound closure under proliferation-restricted conditions, reflecting a combined cellular phenotype involving altered metabolic activity, viable cell mass, adhesion-related features, and cell-cycle-associated regulation. These findings do not establish selective engagement of the migratory machinery or provide conclusive evidence of a migration-exclusive mechanism.

This interpretation is compatible with the broader literature on plasma-induced redox stress. Reactive species generated during CAP treatment can alter processes involved in adhesion, cytoskeletal remodeling, and motility. Our experiments were not designed to follow these signaling events directly, however, and no functional perturbation of individual pathways was performed. For this reason, the observed phenotype should be regarded as a reproducible cellular response to CAP rather than evidence that a particular intracellular pathway is responsible for the reduction in wound closure.

Previous studies in OSCC provide useful context for this interpretation. Kang et al. reported that non-thermal atmospheric-pressure plasma reduced both migration and invasion in oral squamous carcinoma cells and that these effects were accompanied by lower focal adhesion kinase (FAK) expression and reduced MMP-2 and MMP-9 activity [22]. Chang et al. subsequently showed that non-thermal plasma combined with cetuximab inhibited migration and invasion in cetuximab-resistant OSCC cells. In that model, treatment was associated with increased E-cadherin and decreased expression of vimentin, Snail, Slug, MMP-2, and MMP-9 [23]. More recently, Pavlović et al. demonstrated that both direct CAP exposure and plasma-treated medium reduced viability, adhesion, and migration in SCC-25 cells, with antitumor effects also observed in three-dimensional OSCC spheroids [24]. These studies provide relevant context for the wound-closure phenotype observed in our experiments and place it within an emerging body of evidence linking plasma exposure to invasion-related cellular behavior. Direct comparisons should nevertheless be made carefully, as plasma source, working gas, exposure duration, treatment geometry, device design, and cellular model vary considerably between studies, and the experimental contribution of cell proliferation to scratch closure may also differ across protocols. To place our experimental observations in a broader molecular context, we also examined publicly available baseline transcriptomic data for HSC-3 and CAL27 cells. An important distinction is that these data were generated independently of the CAP experiments and therefore provide no information on CAP-induced transcriptional changes. The Pathview/KEGG analysis should instead be viewed as a molecular snapshot of the two cell models before treatment. It shows that both HSC-3 and CAL27 express components of adhesion, extracellular-matrix, focal-adhesion, Rho-family, and actin-cytoskeleton networks that are relevant to migratory behavior [25]. The analysis is therefore useful for understanding the biological background in which the CAP-associated phenotype occurs, but it does not establish that any of these pathways are activated, inhibited, or otherwise directly involved in the response to plasma.

The changes in cadherin expression deserve particular consideration. E-cadherin and N-cadherin were examined because alterations in cell–cell adhesion frequently accompany changes in migratory behavior [26]. Following CAP exposure, E-cadherin increased in both cell lines, whereas N-cadherin levels remained low and did not show a statistically significant change following CAP exposure. An increase in E-cadherin is compatible with stronger epithelial adhesive characteristics and may therefore be relevant to the reduced wound closure observed after CAP treatment. At the same time, the present data are not sufficient to conclude that CAP induces a reversal of epithelial–mesenchymal transition (EMT). Such a conclusion would require a substantially broader panel of epithelial and mesenchymal markers, together with experiments addressing the functional role of these changes.

The E-cadherin findings are also consistent with established observations in OSCC biology. Hashimoto et al. showed that membrane E-cadherin expression decreases at the invasive front of OSCC and that this reduction is associated with tumor dedifferentiation and invasion, whereas N-cadherin-positive cells were relatively uncommon and were not associated with clinicopathological progression [27]. The pattern observed in our experiments (higher E-cadherin after CAP exposure in the presence of persistently low N-cadherin) therefore is consistent with the established association between E-cadherin-mediated cell–cell adhesion and epithelial cell-state characteristics. Plasma-based studies have likewise reported increased E-cadherin together with lower expression of mesenchymal and invasion-associated markers in OSCC models [23]. Even so, our data support only the conclusion that CAP can modulate an epithelial adhesion marker. Whether this change plays a causal role in limiting migration remains an open question.

A second major effect of CAP was the reduction in WST-1 signal and Cyclin A expression [28]. These two findings are related but should not be interpreted as equivalent measures. WST-1 reflects relative cellular metabolic activity and is influenced by both the metabolic state and the number of viable cells; it does not directly measure cell proliferation, viability, or the mode of cell death. Cyclin A, by contrast, is closely linked to S-phase progression and the G2/M transition [18]. Its reduction after CAP exposure is consistent with altered cell-cycle regulation, but a change in a single cell-cycle-associated protein cannot establish either cell-cycle arrest or the specific phase involved. Direct cell counting, DNA-synthesis assays, flow-cytometric analysis of cell-cycle distribution, and evaluation of additional cyclins and cyclin-dependent kinase regulators would help clarify this aspect of the response. Previous studies have reported CAP-associated alterations in cell-cycle regulation in oral and head and neck cancer models. Chang et al. observed DNA damage, apoptosis, and accumulation of cells in the sub-G1 fraction after non-thermal atmospheric-pressure plasma treatment of oral squamous carcinoma cells, with evidence pointing to involvement of the ATM/p53 pathway [29]. Welz et al. similarly described a treatment-time-dependent loss of viability, DNA fragmentation, and apoptosis in HNSCC cell lines exposed to CAP [30]. More recently, CAP-activated medium was shown to alter cell-cycle distribution and induce apoptosis in HSC-3 and other head and neck cancer cells [31]. Our finding of reduced Cyclin A expression is consistent with this broader literature but should be interpreted as evidence of altered expression of a cell-cycle-associated regulatory protein rather than as direct evidence of cell-cycle arrest at a defined phase. The possibility that CAP preferentially affects malignant cells is especially relevant from a translational perspective. Lee et al. reported greater susceptibility of OSCC cells to CAP than normal human gingival fibroblasts and associated this difference with plasma-induced dysfunction of overexpressed EGFR [32]. Di Giacomo et al. also identified treatment conditions under which CAP-activated medium inhibited HSC-3 and other head and neck cancer cells while exerting more limited effects on non-neoplastic keratinocytes [31]. These observations raise the possibility of a therapeutic window. Its existence, however, is likely to depend on several factors, including the plasma device, dose, exposure conditions, medium composition, and biological model. Since we did not include a non-neoplastic oral-cell comparator, our experiments cannot address whether the treatment conditions used here were tumor-selective.

Several limitations should therefore be kept in mind when interpreting the present results. First, the experiments were performed in only two HNSCC cell lines and under two-dimensional culture conditions. Confirmation in additional OSCC models, three-dimensional systems, and ultimately in vivo models will be needed to determine how broadly the findings apply. Second, the lack of a non-neoplastic oral reference model limits any interpretation regarding tumor selectivity or the existence of a therapeutic window. Direct side-by-side comparisons with appropriate non-malignant oral cell models will therefore be necessary in future studies. Moreover, the lack of a specific sham/device-off airflow control is a technical limitation of the current work. Although the short exposure duration, fixed 10 mm treatment distance, standardized medium volume, and limited temperature variation reduce the likelihood that thermal or gross mechanical effects fully account for the observed phenotype, subtle physical effects associated with device operation cannot be completely excluded. Future experiments should therefore include dedicated sham-airflow controls to distinguish plasma-mediated biological effects from potential mechanical effects related to gas flow or device operation.

Although Ara-C was used during the wound-healing assay to minimize proliferation, the simultaneous reduction in WST-1 signal indicates that changes in overall cellular fitness may still have influenced wound closure. Accordingly, the present results do not establish a migration-specific inhibitory mechanism.

This study’s absence of direct cell death and cytotoxicity mode discrimination testing is another important limitation. Although CAP exposure resulted in a significant decrease in WST-1 absorbance, this readout cannot distinguish among metabolic suppression, reduced viable cell mass, altered proliferation, apoptosis, or necrotic cell death. Therefore, firm conclusions regarding the precise mechanism or extent of CAP-associated cytotoxicity cannot be drawn from the present dataset. Future studies incorporating multiparametric flow-cytometric apoptosis and necrosis assays, together with complementary biochemical markers, will be required to define the cellular responses induced by CAP more precisely. The transcriptomic component of the study is similarly limited by its descriptive nature: the DepMap data represent baseline expression and were not accompanied by functional pathway perturbation. Consequently, these analyses provide molecular context but cannot identify the pathways responsible for the CAP-associated phenotype.

Finally, the absence of direct intracellular ROS/RNS measurements represents an additional limitation. CAP-derived reactive species are widely considered important mediators of plasma-induced biological responses; however, their intracellular generation and contribution to the effects observed here were not experimentally assessed. Direct redox profiling, together with antioxidant or scavenger-based rescue experiments, will therefore be required to determine whether ROS/RNS contribute causally to the observed cellular phenotype.

Overall, despite these limitations, the present study provides a controlled in vitro characterization of the response of two biologically distinct OSCC models to a standardized 20 s CAP exposure. CAP treatment was associated with delayed wound closure under proliferation-restricted conditions and with a reduced WST-1 signal in both cell lines, together with increased E-cadherin and reduced Cyclin A expression. These findings support further investigation of CAP as a modulator of OSCC cellular fitness, cell–cell adhesion, and growth-associated regulatory pathways.

The baseline transcriptomic analysis provides an additional molecular framework for interpreting these cellular responses by showing that the two models express a broad repertoire of adhesion- and cytoskeleton-related genes [33,34]. It does not, however, identify the mechanism through which CAP produces the observed phenotype. Future experiments combining normal oral-cell controls, sham treatment, complementary migration and invasion assays, direct assessment of proliferation and cell-cycle distribution, multi-parameter flow cytometry, direct redox profiling, and targeted mechanistic approaches should help determine which of these responses are primary consequences of plasma exposure and which arise secondarily from changes in cell viability or metabolic state.

## 5. Conclusions

In conclusion, this in vitro study shows that a standardized 20 s CAP exposure was associated with consistent biological responses across two distinct OSCC cell models, CAL27 and HSC-3, derived from primary and metastatic lesions, respectively. These responses included slower wound closure under proliferation-restricted conditions, a reduced WST-1 signal, lower Cyclin A expression, and increased E-cadherin levels, whereas N-cadherin levels remained low and did not show a statistically significant change. Because intracellular ROS/RNS were not directly measured and a dedicated sham airflow control was not included, the specific contribution of plasma-generated reactive species and other physical components of CAP to the observed phenotype cannot be established. The present findings therefore support the interpretation that short-term CAP exposure is associated with a broader cellular response in OSCC models, involving changes in relative metabolic activity/viable cell mass, cell–cell adhesion, and the expression of a cell-cycle-associated regulatory protein, rather than demonstrating a single, isolated anti-migratory mechanism, direct cytotoxic effect, or defined cell-cycle arrest.

Baseline transcriptomic data provided additional molecular context by showing differences between HSC-3 and CAL27 in the expression of genes related to focal adhesion, Rho-family signaling, and actin-cytoskeleton organization. However, because these datasets represent untreated baseline states, they do not demonstrate a CAP-dependent molecular mechanism or pathway activation.

Taken together, these findings provide an initial in vitro characterization of the response of two biologically distinct OSCC models to a standardized CAP exposure. The present study does not address clinical tumor selectivity, the precise role of intracellular ROS/RNS, the specific mode of cell death, or definitive cell-cycle effects, as non-neoplastic oral cells, direct redox measurements, cell-cycle profiling, and apoptosis/necrosis assays were not included.

Further investigation should therefore incorporate appropriate non-neoplastic oral-cell comparators, sham airflow controls, direct ROS/RNS measurements, complementary proliferation and cell-cycle analyses, and direct assessment of cell death. Validation in additional cell models, three-dimensional systems, and ultimately in vivo models will also be necessary to clarify the underlying mechanisms, potential selectivity, and translational relevance of CAP in OSCC.

## Data Availability

All data supporting the findings presented in this study are included in the article. Additional study materials are available from the corresponding author upon reasonable request.

## References

[1] Chen Z., Chen G., Fang T. (2025). Advances in cold atmospheric plasma therapy for cancer. MedComm.

[2] Stańczyk B., Wiśniewski M. (2024). The promising potential of cold atmospheric plasma therapies. Plasma.

[3] Babajani A., Eftekharinasab A., Bekeschus S., Mehdian H., Vakhshiteh F., Madjd Z. (2024). Reactive oxygen species from non-thermal gas plasma (CAP): Implication for targeting cancer stem cells. Cancer Cell Int..

[4] Khalaf A.T., Abdalla A.N., Ren K., Liu X. (2024). Cold atmospheric plasma (CAP): A revolutionary approach in dermatology and skincare. Eur. J. Med. Res..

[5] Liu S., Liu J., Wang Y., Deng F., Deng Z. (2025). Oxidative stress: Signaling pathways, biological functions, and disease. MedComm.

[6] Wang W., Zheng P., Yan L., Chen X., Wang Z., Liu Q. (2024). Mechanism of non-thermal atmospheric plasma in anti-tumor: Influencing intracellular RONS and regulating signaling pathways. Free Radic. Res..

[7] Glorieux C., Liu S., Trachootham D., Huang P. (2024). Targeting ROS in cancer: Rationale and strategies. Nat. Rev. Drug Discov..

[8] Matthews H.K., Bertoli C., de Bruin R.A.M. (2022). Cell cycle control in cancer. Nat. Rev. Mol. Cell Biol..

[9] Hydbring P., Malumbres M., Sicinski P. (2022). Non-canonical functions of cell cycle cyclins and cyclin-dependent kinases. Nat. Rev. Mol. Cell Biol..

[10] Ridley A.J. (2023). Rho GTPase signalling in cell migration. Nat. Rev. Mol. Cell Biol..

[11] Sies H., Jones D.P. (2020). Reactive oxygen species (ROS) as pleiotropic physiological signalling agents. Nat. Rev. Mol. Cell Biol..

[12] Cascardi E., Della Mura M., Sgarro N., Minei S., Cazzato G., Maiorano E., Lo Muzio L., Bizzoca M.E., Vieira E Silva F.F., Lo Muzio E. (2026). Chorus line in oral squamous cell carcinoma: How stromal and immune players orchestrate tumor progression (Review). Int. J. Mol. Med..

[13] Mudianto T., Campbell K.M., Webb J., Zolkind P., Skidmore Z.L., Riley R., Barnell E.K., Ozgenc I., Giri T., Dunn G.P. (2021). Yap1 Mediates Trametinib Resistance in Head and Neck Squa-mous Cell Carcinomas. Clin. Cancer Res..

[14] Vardhan N.R., Preetha K., Bonney L.J., Ranganadha R.A. (2025). Expression Dynamics of Metastasis-Associated Markers in Oral Squamous Cell Carcinoma Cell Lines CAL27 and HSC-3. Res. J. Biotechnol..

[15] Sun Y., Zboril E.K., De La Chapa J.J., Chai X., Da Conceicao V.N., Valdez M.C., McHardy S.F., Gonzales C.B., Singh B.B. (2022). Inhibition of Ca^2+^ entry by capsazepine analog CIDD-99 prevents oral squamous carcinoma cell proliferation. Front. Physiol..

[16] Sorrentino C., Lauretta C., D’Angiolo R., Musella S., Giovannelli P., Bertamino A., Ostacolo C., Monterrey I.G., Migliaccio A., Castoria G. (2026). Rewiring melanoma cell fate: TRPM8 modulators trigger apoptosis and boost NK cell cytotoxicity. Cell Death Dis..

[17] Di Donato M., Cristiani C.M., Capone M., Garofalo C., Madonna G., Passacatini L.C., Ottaviano M., Ascierto P.A., Auricchio F., Carbone E. (2025). Role of the androgen receptor in melanoma aggressiveness. Cell Death Dis..

[18] Kanehisa M., Goto S. (2000). KEGG: Kyoto encyclopedia of genes and genomes. Nucleic Acids Res..

[19] Hua D., Cai D., Ning M., Yu L., Zhang Z., Han P., Dai X. (2021). Cold atmospheric plasma selectively induces G0/G1 cell cycle arrest and apoptosis in AR-independent prostate cancer cells. J. Cancer.

[20] Colapietra F., Della Monica P., Di Napoli R., França Vieira E., Silva F., Settembre G., Marino M.M., Ballini A., Cantore S., Di Domenico M. (2025). Epigenetic modifications as novel biomarkers for diagnosis, prognosis, and therapeutic targeting in thyroid, pancreas, and lung neuroendocrine tumors. J. Clin. Med..

[21] Zhang S., Yuan L., Lin P., Yang G., Zhou X., Xu J., Wu M., Huang Y. (2026). Cancer neuroscience: Signaling pathways and new therapeutic strategies for cancer. Signal Transduct. Target. Ther..

[22] Kang S.U., Seo S.J., Kim Y.S., Shin Y.S., Koh Y.W., Lee C.M., Yang S.S., Lee J.S., Moon E., Kang H. (2017). Comparative Effects of Non-Thermal Atmospheric Pressure Plasma on Migration and Invasion in Oral Squamous Cell Cancer, by Gas Type. Yonsei Med. J..

[23] Chang J.W., Kang S.U., Shin Y.S., Seo S.J., Kim Y.S., Yang S.S., Lee J.S., Moon E., Baek S.J., Kim C.H. (2015). Combination of NTP with cetuximab inhibited invasion/migration of cetuxi-mab-resistant OSCC cells: Involvement of NF-kB signaling. Sci. Rep..

[24] Pavlović O., Lazarević M., Jakovljević A., Škoro N., Puač N., Mojsilović S., Miletić M. (2025). Antitumor Potential of Different Treatment Approaches Using Cold Atmospheric Pressure Plasma on Oral Squamous Cell Carcinoma Models: In Vitro Study. Biomedicines.

[25] Sun J., Hosen M.B., Deng W.M., Tian A. (2025). Epithelial polarity loss and multilayer formation: Insights into tumor growth and regulatory mechanisms. Bioessays.

[26] Mendonsa A.M., Na T.Y., Gumbiner B.M. (2018). E-cadherin in contact inhibition and cancer. Oncogene.

[27] Hashimoto T., Soeno Y., Maeda G., Taya Y., Aoba T., Nasu M., Kawashiri S., Imai K. (2012). Progression of Oral Squamous Cell Carcinoma Accompanied with Reduced E-Cadherin Expression but Not Cadherin Switch. PLoS ONE.

[28] Li B., Ming H., Qin S., Nice E.C., Dong J., Du Z., Huang C. (2025). Redox regulation: Mecha-nisms, biology and therapeutic targets in diseases. Signal Transduct. Target. Ther..

[29] Chang J.W., Kang S.U., Shin Y.S., Kim K.I., Seo S.J., Yang S.S., Lee J.S., Moon E., Baek S.J., Lee K. (2014). Non-thermal atmospheric pressure plasma induces apoptosis in oral cavity squamous cell carcinoma: Involvement of DNA-damage-triggering sub-G1 arrest via the ATM/p53 pathway. Arch. Biochem. Biophys..

[30] Welz C., Emmert S., Canis M., Becker S., Baumeister P., Shimizu T., Morfill G.E., Harréus U., Zimmermann J.L. (2015). Cold Atmospheric Plasma: A Promising Complementary Therapy for Squamous Head and Neck Cancer. PLoS ONE.

[31] di Giacomo V., Balaha M., Pinti M., Di Marcantonio M.C., Cela I., Acharya T.R., Kaushik N.K., Choi E.H., Mincione G., Sala G. (2025). Cold atmospheric plasma activated media selectively affects human head and neck cancer cell lines. Oral Dis..

[32] Lee J.H., Om J.Y., Kim Y.H., Kim K.M., Choi E.H., Kim K.N. (2016). Selective Killing Effects of Cold Atmospheric Pressure Plasma with NO Induced Dysfunction of Epidermal Growth Factor Receptor in Oral Squamous Cell Carcinoma. PLoS ONE.

[33] Perrotti V., Caponio V.C.A., Muzio L.L., Choi E.H., Di Marcantonio M.C., Mazzone M., Kaushik N.K., Mincione G. (2022). Open Questions in Cold Atmospheric Plasma Treatment in Head and Neck Cancer: A Systematic Review. Int. J. Mol. Sci..

[34] Maravic T., Petrucci G., Giacomo V.D., Mazzitelli C., Acharya T.R., Kaushik N.K., Choi E.H., Rapino M., D’Urso D., Josic U. (2025). In vitro study of cold atmospheric plasma-induced proliferation inhibition and morphological changes in head and neck carcinoma cell lines. J. Dent..

